# Comparative transcriptome analysis provides insights into molecular mechanisms for parthenocarpic fruit development in eggplant (*Solanum melongena L*.)

**DOI:** 10.1371/journal.pone.0179491

**Published:** 2017-06-12

**Authors:** Xia Chen, Min Zhang, Jie Tan, Shuping Huang, Chunli Wang, Hongyuan Zhang, Taiming Tan

**Affiliations:** Wuhan Vegetable Research Institute, Wuhan Academy of Agricultural Science and Technology, Wuhan, Hubei, China; Instituto de Biologia Molecular y Celular de Plantas, SPAIN

## Abstract

Genetic control of parthenocarpy, a desirable trait in edible fruit with hard seeds, has been extensively studied. However, the molecular mechanism of parthenocarpic fruit development in eggplant (*Solanum melongena L*.) is still unclear. To provide insights into eggplant parthenocarpy, the transcriptomic profiles of a natural parthenocarpic (PP05) and two non-parthenocarpic (PnP05 and GnP05) eggplant lines were analyzed using RNA-sequencing (RNA-seq) technology. These sequences were assembled into 38925 unigenes, of which 22683 had an annotated function and 3419 were predicted as novel genes or from alternative splicing. 4864 and 1592 unigenes that were identified as DEGs between comparison groups PP05 *vs* PnP05 and PP05 *vs* GnP05, respectively. 506 common DEGs were found contained in both comparison groups, including 258 up-regulated and 248 down-regulated genes. Functional enrichment analyses identified many common or specific biological processes and gene set potentially associated with plant development. The most pronounced findings are that differentially regulated genes potentially-related with auxin signaling between parthenocarpic and non-parthenocarpic eggplants, *e*.*g*. calcium-binding protein PBP1 and transcription factor E2FB, which mediate the auxin distribution and auxin-dependent cell division, respectively, are up-regulated in the PP05; whereas homologs of GH3.1 and AUX/IAA, which are involved in inactivation of IAA and interference of auxin signaling, respectively, are down-regulated in PP05. Furthermore, gibberellin and cytokinin signaling genes and genes related to flower development were found differentially regulated between these eggplant lines. The present study provides comprehensive transcriptomic profiles of eggplants with or without parthenocarpic capacity. The information will deepen our understanding of the molecular mechanisms of eggplant parthenocarpy. The DEGs, especially these filtered from PP05 *vs* PnP05 + GnP05, will be valuable for further investigation of key genes involved in the parthenocarpic fruit development and genomics-assisted breeding.

## Introduction

Eggplant (*Solanum melongena L*.), a herbaceous belongs to the genus *Solanum*, is a vegetable crop species of global importance. Eggplant is identified to originate from Africa, and is currently widely cultivated in Asia, Africa, Europe and the Near East [1]. The data from the FAO (Food and Agriculture Organization of the United Nations, http://faostat.fao.org) indicates that the annual global yield of eggplant is 51.3 megatons in 2014. And Asia accounts for over than ninety-percentage of the total eggplant production during the period from 2010 to 2014. Eggplant is one of the most popular vegetable since it is delicious in taste, and is an excellent source of fibers, vitamins, minerals as well as certain polyphenols that exhibit antioxidant activities [2, 3]. Despite the widespread cultivation of eggplant, many influential factors are continuing to cause extensive losses from planting to harvest of eggplant, such as pests, diseases and weeds [4]. Besides, low temperature, which affects the pollination and fertilization, is also a serious constraint to the yield and quality of early-maturing eggplant varieties [5]. To avoid these adverse effects caused by low temperature, spray treatment with plant growth regulators, like 2,4-dichlorophenoxyacetic acid, was applied to the eggplant planting. However, undesirable consequences, such as malformed fruits, loss of flavor and drug residual, which are induced by inappropriate use of the plant growth regulators, would reduce the fruit value and have a potential risk for consumers.

Parthenocarpy, a highly desirable trait in horticultural plant breeding, is characterized as that ovary can development into seedless fruit without pollination and fertilization [6]. Parthenocarpic varieties exhibit tolerance to unfavorable environmental stresses [7], have improved fruit set, yield and quality [8, 9]. Parthenocarpic fruit development can be achieved by many methods, *e*.*g*. selection of natural mutant with parthenocarpic ability [10], creation of parthenocarpic mutant with physical, chemical or genetic strategies [11–13], treatment of flower buds with phytohormones [14], and interspecific hybridization to alter ploidy [15], *etc*. Investigations suggest that the development of parthenocarpic fruit is mediated mainly by auxin and gibberellin (GA), though other factors have also been confirmed to be related with the process of parthenocarpy [16]. For example, higher synthesis of GA in tomato ovaries achieved by parthenocarpic fruit (*pat*) genes mutations leads to parthenocarpy [17], and over-expression of auxin biogenesis gene (*iaaM*) or auxin response gene (*rolB*) are alternative approaches to induce parthenocarpy [18, 19]. Compared to tomato, researches on mechanisms of parthenocarpy and breeding of parthenocarpic cultivars in eggplant are relatively less. Some researches show that parthenocarpic eggplant does not have negative pleiotropic effects as observed in parthenocarpic tomato [20]. Available data suggest that the occurrence of eggplant parthenocarpy is also a complex biological process, which is influenced, at least, by phytohormones, environmental and genetic factors [21].

Breeding of eggplant parthenocarpic cultivars is time-consuming and labor-intensive [20]. Increased understanding of the underlying genetic basis was promised to accelerate the breeding process. Recently, several DNA markers or genetic loci related with eggplant parthenocarpy were identified, such as E75/M53-70 [22], *smpc77* [23], *Cop3*.*1* and *Cop8*.*1* [24]. Analysis of eggplant facultative-parthenocarpic line D-10 and non-parthenocarpic line 03–2 using suppression subtractive hybridization (SSH) strategy identified five parthenocarpy-related ESTs [25]. In another trial using the SSH method, seedless and normal fruits from the facultative-parthenocarpic eggplant D-10 were investigated, and three unigenes, which encoding MADS-box transcription factor, pistil extensin-like protein and fruit-ripening-related protein, were selected as parthenocarpy candidate genes [26]. More elements and mechanisms involved in the parthenocarpic fruit development in eggplant still need to be discovered, and the completion of the draft genome sequence of eggplant [27] provides us a new platform for this purpose. It was presumed that the genetic program for fruit development might be already switched on during early stages of flower development before anthesis [28]. Therefore, transcriptome profiles of flower buds of a parthenocarpic eggplant line PP05, and two non-parthenocarpic eggplant lines PnP05 and GnP05, were analyzed using the next generation sequencing (RNA-seq) technology. Differentially expressed genes between the parthenocarpic and non-parthenocarpic eggplants were screened, so as to comprehensively exploit parthenocarpic genes and bring insight into the mechanisms of parthenocarpy in eggplant.

## Materials and methods

### Plant growth and sampling

PP05, a natural parthenocarpic eggplant line (purple), and two non-parthenocarpic eggplant lines, PnP05 (purple) and GnP05 (green), were used in the present study (S1 Fig). All plant lines were planted in early spring and grown in the greenhouse at Wuhan Vegetable Research Institute, China, separately. The investigation of flower and fruit development was performed with the average daily maximum temperature of 21.6°C and the average daily minimum temperature of 11.6°C. A randomized complete block design with four replicates was used in this study. For each replicate, ten flower buds were collected from at least five different plants at one day before anthesis. Samples were frozen in liquid nitrogen and kept at minus 70°C until use.

### RNA isolation and library preparation for transcriptome sequencing

The RNA preparation, cDNA libraries construction and Illumina sequencing were performed in Novogene (Beijing, China). Total RNA was extracted from freshly frozen flower buds using the Trizol reagent (Invitrogen, Carlsbad, CA, USA) and purified using the RNeasy Plant Mini kit (Qiagen, Valencia, CA, USA) according to the manufacturers’ instructions. RNA integrity was assessed using the RNA Nano 6000 Assay Kit of the Bioanalyzer 2100 system (Agilent Technologies, CA, USA). Then a total amount of 3 μg RNA per sample was used as input material for the RNA sample preparations. Sequencing libraries were generated using NEBNext^®^ Ultra^™^ RNA Library Prep Kit for Illumina^®^ (NEB, Ipswitch, MA, USA) following manufacturer’s recommendations and index codes were added to attribute sequences to each sample. Briefly, mRNA was purified from total RNA using poly-T oligo-attached magnetic beads. Fragmentation was carried out using divalent cations under elevated temperature in NEBNext First Strand Synthesis Reaction Buffer (5X). First strand cDNA was synthesized using random hexamer primer and M-MuLV Reverse Transcriptase (RNase H). Second strand cDNA synthesis was subsequently performed using DNA Polymerase I and RNase H. Remaining overhangs were converted into blunt ends *via* exonuclease/polymerase activities. After adenylation of 3’ ends of DNA fragments, NEBNext Adaptor with hairpin loop structure were ligated to prepare for hybridization. In order to select cDNA fragments of preferentially 150~200 bp in length, the library fragments were purified with AMPure XP system (Beckman Coulter, Beverly, USA). Then 3 μl USER Enzyme (NEB) was used with size-selected, adaptor-ligated cDNA at 37°C for 15 min followed by 5 min at 95°C before PCR. Then PCR was performed with Phusion High-Fidelity DNA polymerase, universal PCR primers and Index (X) Primer. At last, PCR products were purified using AMPure XP system (BeckmanCoulter, Beverly, MA, USA) and library quality was assessed on the Agilent Bioanalyzer 2100 system (Agilent Technologies, Santa Clara, CA, USA). The clustering of the index-coded samples was performed on a cBot Cluster Generation System using TruSeq PE Cluster Kit v3-cBot-HS (Illumina, San Diego, CA, USA) according to the manufacturer’s instructions. After cluster generation, the library preparations were sequenced on an Illumina Hiseq platform and 125 bp/150 bp paired-end reads were generated.

### Quality control for raw sequencing data and reads mapping to the reference genome

After sequencing, the raw data of FASTQ format were first processed through in-house Perl scripts. In this step, the data with clean reads were obtained by trimming the adapter contaminants, filtering low quality reads and reads containing poly-N. At the same time, Q20-, Q30-scores and GC content of the clean data were calculated. All the downstream analyses were based on the clean reads with high quality.

Data from the “Eggplant Genome Database” were used for mapping. TopHat2 (version 2.1.0) [29] was used to map these paired-end clean reads to the reference genome.

### Prediction of novel transcript and analysis of alternative splicing event

The Cufflinks (version 2.1.1) [30] Reference Annotation Based Transcript assembly method was used to construct and identify both known and novel transcripts from TopHat alignment results. Alternative splicing (AS) events were classified to 12 types by the software ASprofile (version 1.0) [31]. The number of AS events in each sample was estimated, separately.

### Quantification of gene expression levels and analysis of differential expression

The reads mapped to each gene were counted by HTSeq software (version0.5.4, http://www-huber.embl.de/users/anders/HTSeq/index.html). The resulting read count for each gene was normalized with FPKM (expected number of Fragments Per Kilobase of transcript sequence per Millions base pairs sequenced) to measure its expression level. Effects of sequencing depth and gene length for the read counts were considered during the analysis of gene expression levels [30].

Selection of the differentially expressed genes (DEGs) between assigned libraries (PP05 *vs* PnP05; PP05 *vs* GnP05; PP05 *vs* PnP05 + GnP05) was performed using the DESeq R package (version 1.12.0) [32]. The resulting *P*-values were adjusted using Benjamini and Hochberg’s approaches [33] for control of the false discovery rate, and genes with adjusted *P*-value < 0.05 were considered as differentially expressed. Here, the threshold for significant difference in gene expression was set as absolute value of log2 fold change > 1 and adjusted *P*-value < 0.05.

The filtered DEGs were mapped to GO (Gene Ontology) and KEGG (the Kyoto Encyclopedia of Genes and Genomes) database using GOseq R package (release 2.12) [34] and KOBAS (version 2.0) [35] to analyze the significantly enriched GO terms and KEGG pathways, respectively. The false discovery rates were controlled using the Benjamini and Hochberg’s methods [33]. Categories with FDR-adjusted *P*-value < 0.05 were considered as significantly enriched.

### Reverse-transcription real-time quantitative PCR (qRT-PCR)

A total of nine genes were selected randomly for RNA-seq data validation using qRT-PCR (S1 Table). Total RNA was extracted from eggplant samples as described above. First-strand cDNA synthesis was performed in a 20 μl system containing 1 μg total RNA, 1 μl oligo (dT) 18 primer (100 μM), 4.0 μl buffer (5X), 0.4 μl dNTP (25 mM each dNTP), 0.5 μl RNase inhibitor and 0.5 μl M-MuLV reverse transcriptase (200 U/μl, Takara, Dalian, China), and RNase free water to a 20 μl volume. Reactions lacking reverse transcriptase were performed in parallel as control for possible DNA contamination. First-strand cDNA from each reaction was subjected to 5-fold dilution and used in subsequent qPCR. Primers used for qPCR assays are listed in S1 Table. qPCR was prepared in reactions containing 10 μl 2 × SYBR Green real-time PCR Master Mix (Takara), 1 μl diluted first-strand cDNA, 1 μl each PCR primer (2.5 mM) and distilled water to a 20 μl volume. An ABI 7500 fast real-time PCR system (Applied Biosystem, Foster City, CA) was used. The thermal cycle was set as follows: 95°C for 30 sec, then 45 cycles of 95°C for 10 sec and 60°C for 20 sec, and 95°C for 15 min. The qRT-PCR was performed in triplicates. The tubulin gamma (Sme2.5_03686.1_g00005.1), which showed stable expression levels, was selected as internal control. The 2^-ΔΔCt^ method was used for calculation of relative expression levels of target genes [36].

## Results

### An overview of the RNA-seq data

To understand the potential molecular mechanisms involved in parthenocarpic fruit development, three eggplant lines were planted in early spring and investigated under a relatively low temperature. Stigmas were removed at bud stage to analyze the fruit set and evaluate the parthenocarpic capacity. Results revealed that PP05 developed seedless fruits with normal shape and size 20 days after anthesis, with a relatively high fruit set of 93%; whereas PnP05 and GnP05 were unable to develop into mature fruit, indicating that PP05 is natural parthenocarpic, while PnP05 and GnP05 are non-parthenocarpic.

**Fig 1:**
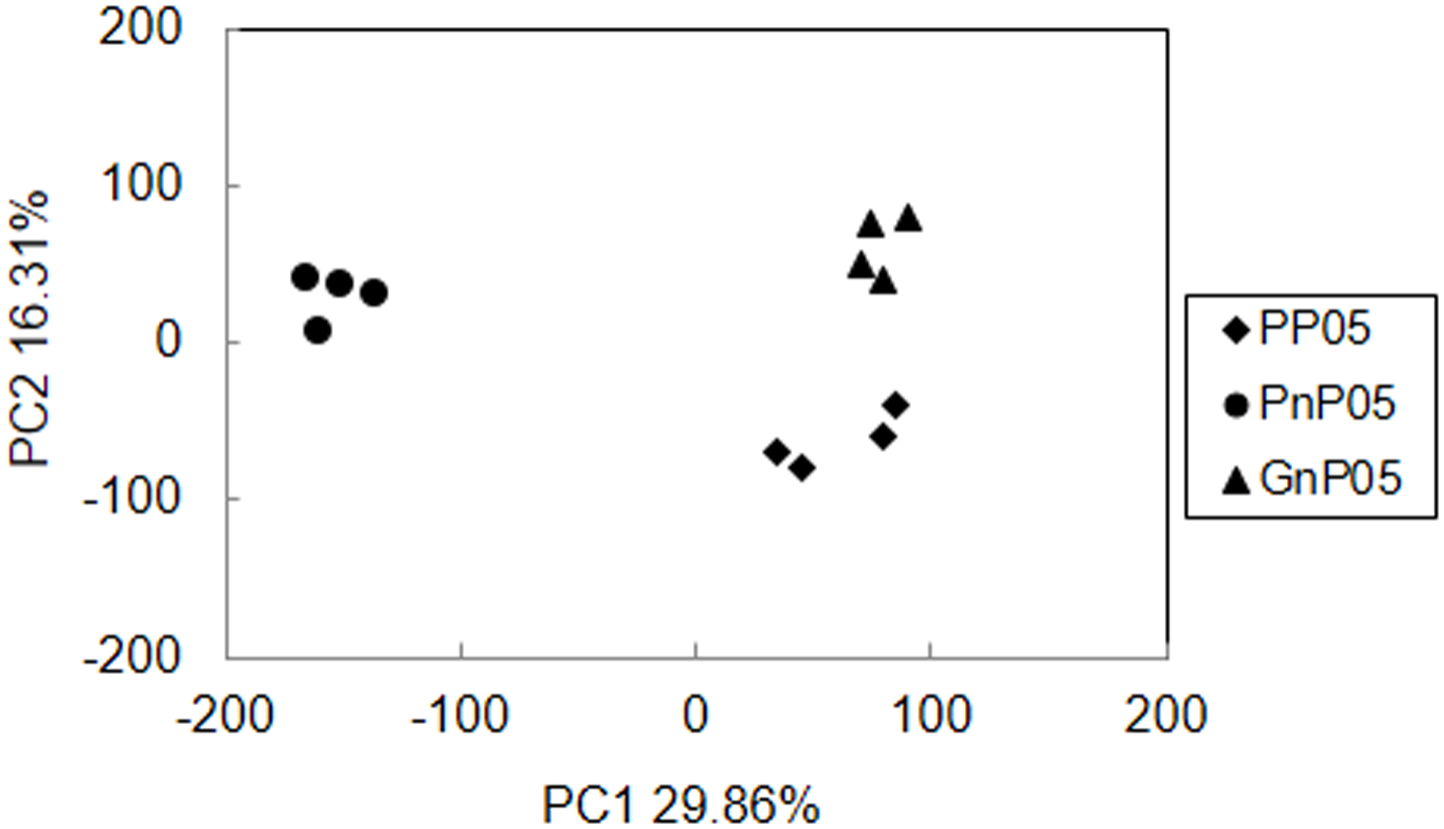
Principal component analysis of four replicates of three eggplant PP05, PnP05 and GnP05.

To investigate the expression patterns of genes in these three eggplant lines, the RNA extracted from the flower buds one day before anthesis were sequenced by Illumina Hiseq 2000 platform. After RNA sequencing, we assessed the quality of the data. An overview of the sequencing and assembly is outlined in Table 1. The Q20, Q30 and GC content in the clean data were calculated and shown in Table 1 and S2 Fig. After removal of adaptor sequences and low-quality reads, an average of 56411205 high-quality clean reads (57775848, and 97.6% of the raw data) was received. And over than 92.2% of the clean reads had Phred-like quality scores at the Q30 level, the average GC content of these samples was 42.5%. Alignments between reads and the reference genome are listed in S2 Table. In the PP05, PnP05 and GnP05 samples, respectively, 79.0%, 80.2% and 80.5% of the total reads from RNA-seq data were mapped uniquely to the genome, while small proportions (less than 3%) were mapped multiply to the reference genome. Mapping of clean reads to exon, intron and intergenic regions of the reference genome are presented in S3 Fig, the average percent of exon reads in total mapped reads of PP05, PnP05 and GnP05, were 84.7%, 83.7% and 84.4%, respectively. The principal components analysis (PCA) were used to analyze the RNA-seq results. These three eggplant lines formed three different clusters, but the four replicates of each eggplant line were found clustered together (Fig 1). Besides, the Pearson correlations between replicates are shown in S3 Table, with R^2^-value > 0.93 among technical replicates in each eggplant line. Alternative splicing (AS) events were analyzed using Cufflinks 2.1.1 software and results were listed in S4 Fig. The most predominant AS types are TSS (alternative 5’ first exon) and TTS (alternative 3’ last exon).

**Table 1:** Major characteristics of RNA-seq data of parthenocarpic and non-parthenocarpic eggplants.

No.	Sample name	Raw reads	Clean reads	Clean bases	Error rate (%)	Q20 (%) ^a^	Q30 (%) ^a^	GC content (%) ^b^
1	PP05_1	59468466	58277782	8.74 G	0.01	97.16	93.16	42.60
2	PP05_2	62713496	60525246	9.08 G	0.02	96.83	92.44	42.64
3	PP05_3	56622724	55143180	8.27 G	0.01	97.26	93.35	42.79
4	PP05_4	50182638	49119262	7.37 G	0.02	96.71	92.20	42.53
5	PnP05_1	63034504	61456426	9.22 G	0.02	96.85	92.35	42.18
6	PnP05_2	47888102	46924756	7.04 G	0.01	97.12	92.92	42.27
7	PnP05_3	66001460	64580670	9.69 G	0.01	97.34	93.41	42.46
8	PnP05_4	53126670	51941728	7.79 G	0.01	97.11	92.91	42.20
9	GnP05_1	54237214	52944902	7.94 G	0.01	96.98	92.73	42.57
10	GnP05_2	61911884	60462982	9.07 G	0.01	97.47	93.71	42.51
11	GnP05_3	61121302	59656812	8.95 G	0.01	97.31	93.51	42.43
12	GnP05_4	57001716	55900718	8.39 G	0.01	97.44	93.67	42.38

Notes:

^*a*^ Q20 (%) and Q30 (%) are the percentages of reads with Phred quality scores over than 20 and 30, respectively.

^*b*^ GC content (%) means G + C bases as the percentage of total bases.

### Differential expression analysis

**Fig 2:**
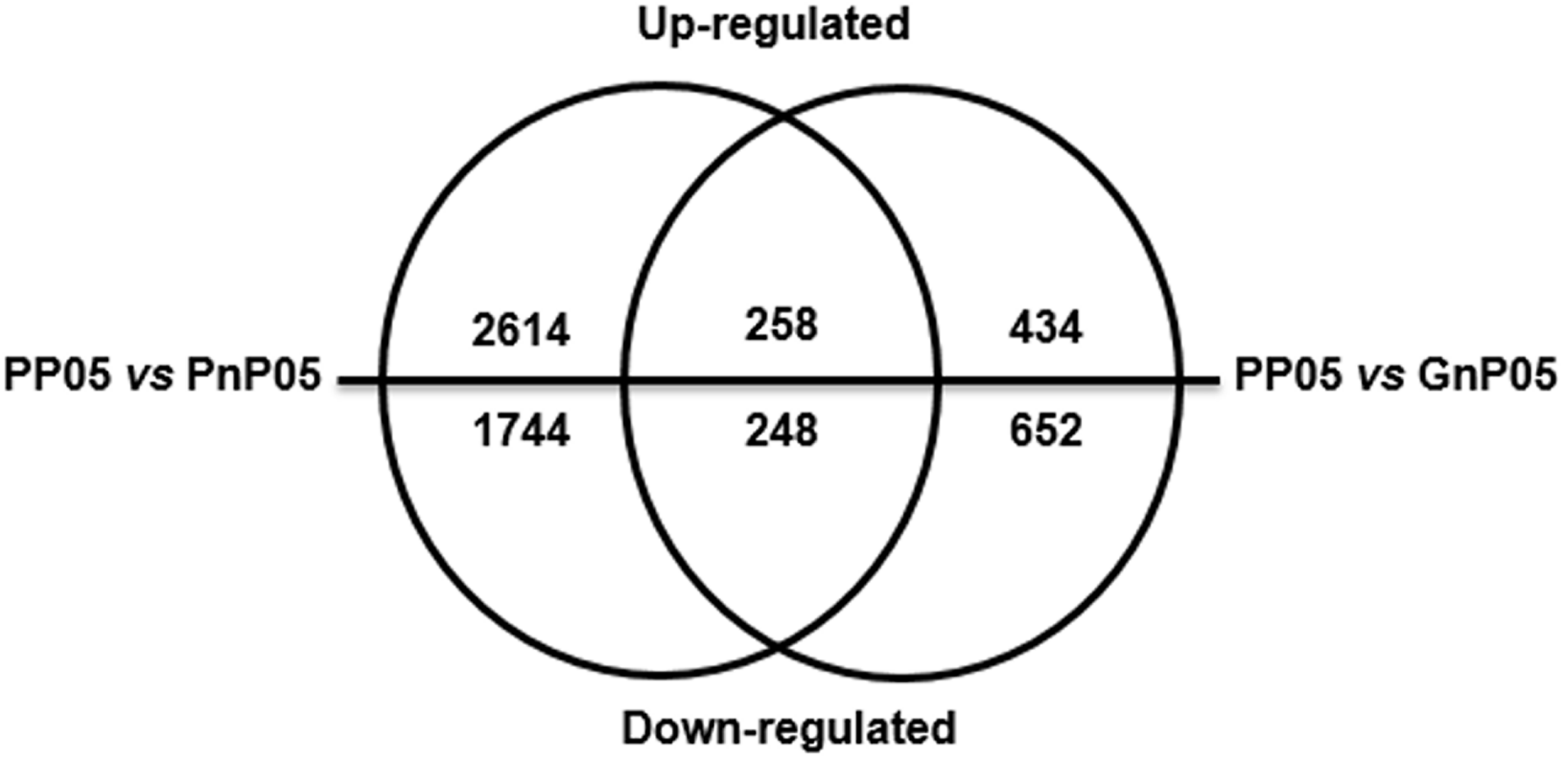
Venn diagram depicting the numbers of DEGs found in these three eggplant lines.

Transcription abundances for mapped genes were calculated according to the methods as described previously [30]. Moreover, differentially expressed genes (DEGs) were identified through pairwise comparison between the parthenocarpic and non-parthenocarpic eggplant libraries, by setting a threshold of absolute value of log2 fold change > 1 and adjusted *P*-value < 0.05. DEGs in PP05 *vs* PnP05 and PP05 *vs* GnP05 were screened. It seems that the pigmentation similarity does not correlate to the differences of parthenocarpy. The results showed that a large number of DEGs were obtained: PP05 *vs* PnP05, 4864 (2872 up- and 1992 down-regulated); while the number of DEGs was 1592 (692 up- and 900 down-regulated) from PP05 *vs* GnP05 (Fig 2). Besides, we noticed that there was also a large number of DEGs between the two non-parthenocarpic lines PnP05 and GnP05 (data not shown). This observation may be caused by the genetic background differences. Therefore, co-regulated genes in both comparison groups (PP05 *vs* PnP05 + GnP05) were further filtered. Finally, a total of 506 commonly regulated DEGs (258 up- and 248-down regulated) were identified (Fig 2) and further investigated. Detailed information of these common DEGs are listed in S3 Table.

### GO classification of DEGs

**Fig 3:**
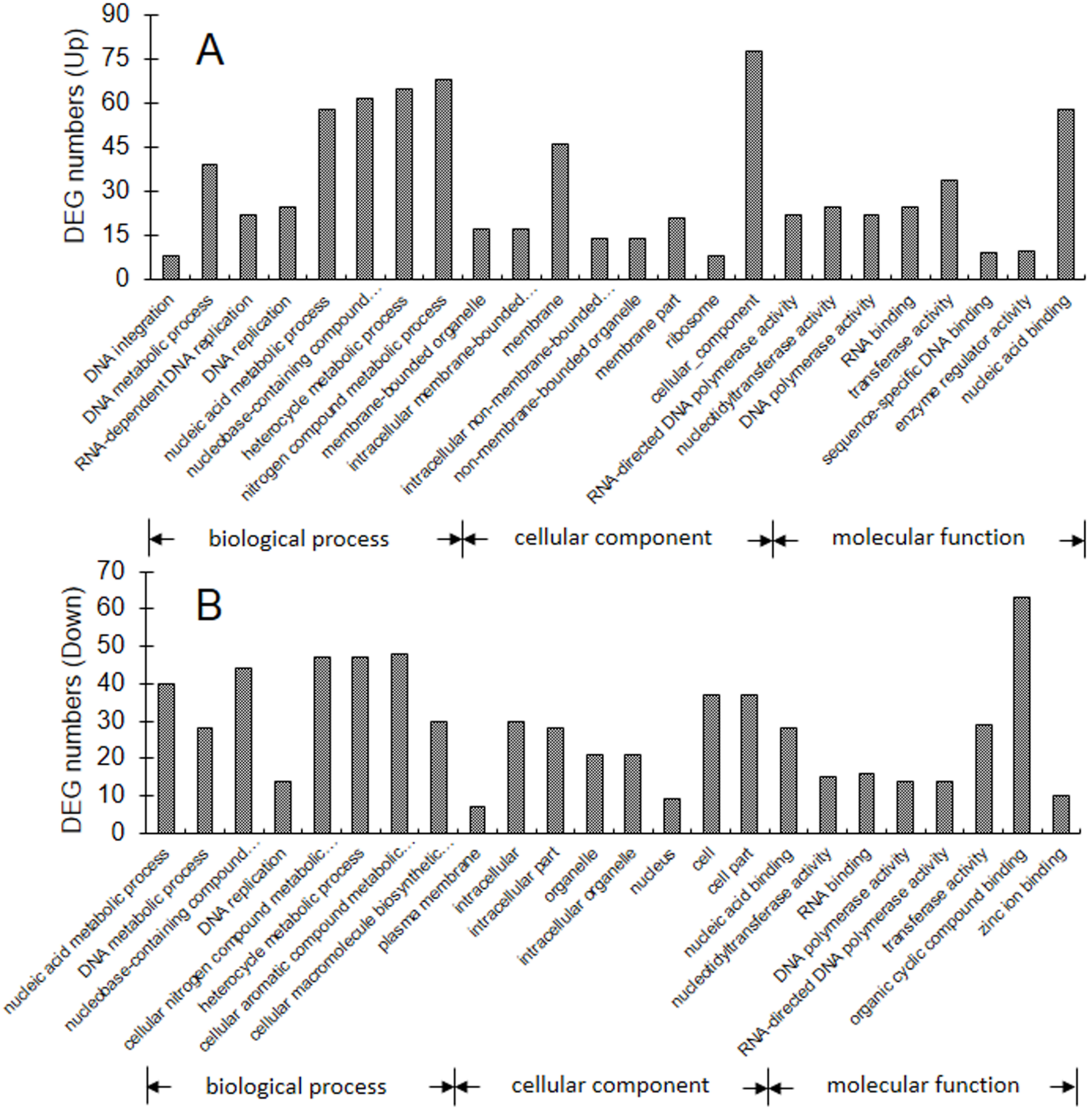
Histograms showing GO functional analysis of the up-regulated (A) and down-regulated (B) common DEGs. The *x*-axis indicates the names of the eight most abundant classes in each of the three main GO categories. The *y*-axis indicates the numbers of common DEGs.

To facilitate the global analysis of gene expression, the functional classes of commonly regulated DEGs (PP05 *vs* PnP05 + GnP05) were subjected to GO enrichment analysis with the GOseq R package. Common DEGs were categorized into 1255 functional groups that were clustered in three main categories of the GO classification “biological process” (BF), “cellular component” (CC) and “molecular function” (MF). The most enriched GO terms of the up- and down-regulated common DEGs in each main GO category are shown in Fig 3 and S5 Table. For the up-regulated DEGs, DNA integration (GO: 0015074, with 8 genes), membrane-bounded organelle (GO: 0043227, with 17 genes), and RNA-directed DNA polymerase activity (GO: 0003964, with 22 genes) were most enriched in the categories of BP, CC and MF, respectively. While for the down-regulated DEGs, nucleic acid metabolic process (GO: 0090304, with 40 genes, plasma membrane (GO: 0005886, with 7 genes), and nucleic acid binding (GO: 0003676, with 28 genes), respectively, were dominant in the categories of BP, CC and MF.

### KEGG enrichment of DEGs

**Fig 4:**
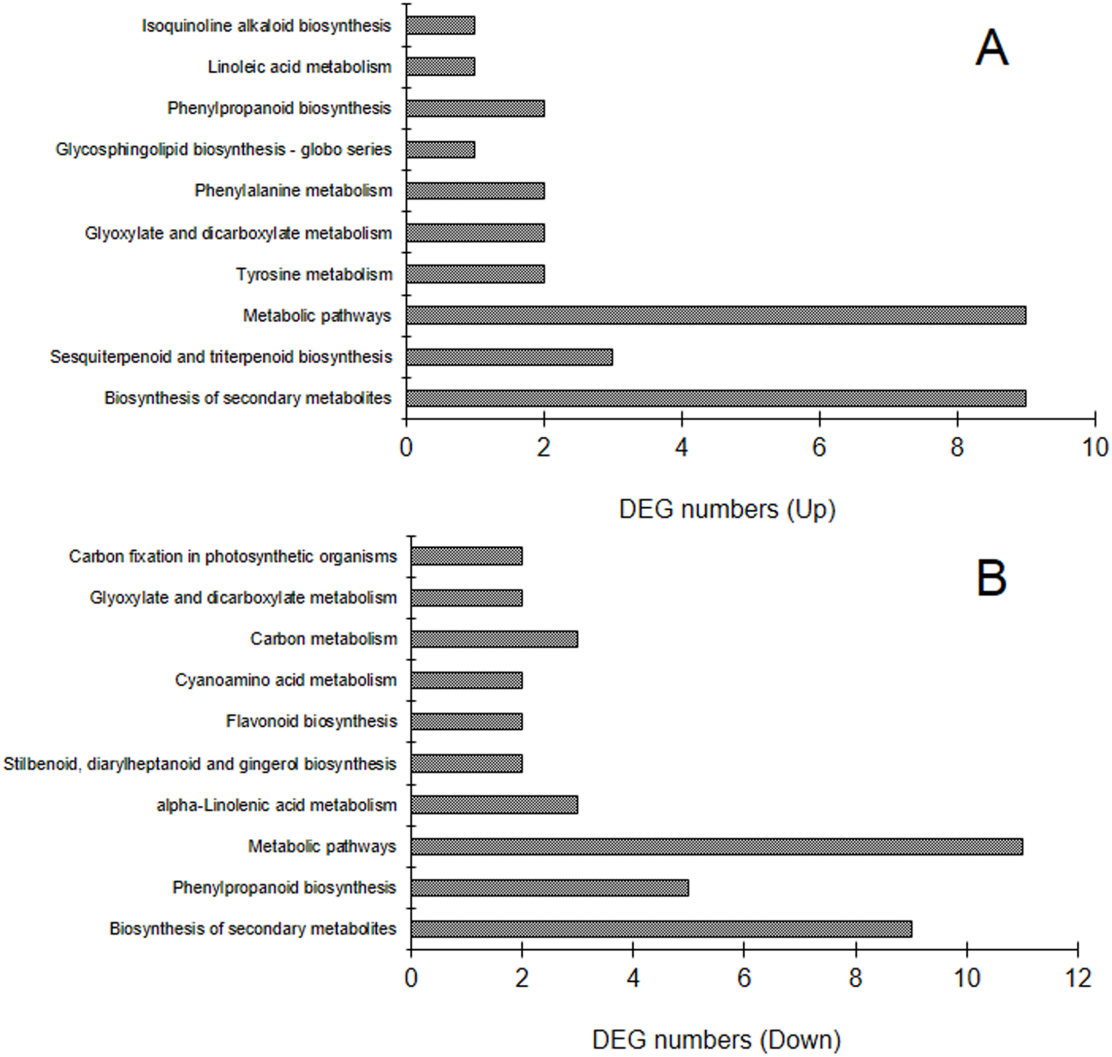
Statistics of KEGG enrichment of the up-regulated (A) and down-regulated (B) common DEGs. Histograms indicate 10 most abundant KEGG pathways.

To identify the biological pathways that are active in the parthenocarpic eggplant, the detected genes were mapped to the reference pathways in the KEGG database and compared them with the whole transcriptome background, with a view of searching genes involved in metabolic or signal transduction pathways that were significantly enriched. As shown in Fig 4 and S6 Table, pathways with the greatest representation of the up-regulated DEGs were “biosynthesis of secondary metabolites” (sly01100, with 9 members), “sesquiterpenoid and triterpenoid biosynthesis” (sly00909, with 3 members) and “metabolic pathways” (sly01100, with 9 members) (Fig 4A). While “biosynthesis of secondary metabolites” (with 9 members), “metabolic pathways” (with 11 members) and “phenylpropanoid biosynthesis” (sly00940, with 5 members) were the most abundant KEGG pathways of the down-regulated DEGs (Fig 4B). These annotations provide a resource for investigation of specific processes, functions and pathways of eggplant fruit set.

### Expression analysis of eggplant parthenocarpy-related genes

Among these commonly regulated DEGs, genes predicted to participate in plant hormone signal transduction were identified. Auxin signaling pathway, which is regarded as a key regulator of fruit growth, development and parthenocarpy, as well as many other physiological processes, are found in these DEGs, such as these encoding CYP83B1, calcium-binding protein PBP1, transcription factor E2FB-like, and F-box proteins, *etc* (Table 2). Besides, several genes encoding proteins related to gibberellin (GA) and cytokinin (CTK) signaling were differentially transcribed in PP05 *vs* PnP05 + GnP05, like serine/threonine-protein kinase SAPK2, gibberellin 3-beta-dioxygenase and cytokinin hydroxylases (Table 2). In addition, we found many common DEGs functionally related to the regulation of flower development, which may be relevant to parthenocarpy, such as NAC domain-containing proteins, AGAMOUS-like MADS-box protein, zinc finger protein CONSTANS-LIKE 2-like, and so on (Table 2). Other relevant groups of proteins encoded by common DEGs, including signaling of other plant hormones, regulation of secondary metabolism, photosynthesis, as well as cellular structure were also identified (S4 Table). Notably, about 47% common DEGs were unknown or unannotated genes, they may also contribute to the parthenocarpic fruit development in eggplant.

**Table 2:** A list of common DEGs potentially related with eggplant parthenocarpy.

Gene ID	Average expression level ^a^			(log2) fold change		Regulation	KEGG annotation
	PP05	PnP05	GnP05	PP05/PnP05	PP05/GnP05		
Genes related to auxin-signaling							
Sme2.5_00989.1_g00006.1	570.3	61.8	151.8	3.2	1.9	Up	calcium-binding protein PBP1
Sme2.5_00095.1_g00019.1	43.0	5.0	9.5	3.1	2.2	Up	calcium-binding protein PBP1-like
Sme2.5_00885.1_g00011.1	0.3	243.0	1144.0	-9.9	-12.2	Down	serine/threonine-protein phosphatase 6 regulatory ankyrin repeat subunit B
Sme2.5_14564.1_g00002.1	147.8	34	25.3	2.2	2.5	Up	transcription factor E2FB-like
Sme2.5_00079.1_g00011.1	33.0	10.0	8.3	1.8	2.0	Up	F-box protein PP2-B15-like
Novel03544	0	30.0	37.3	--- ^b^	---	Down	F-box protein At3g58530
Sme2.5_01347.1_g00009.1	21.0	55.3	47.0	-1.4	-1.2	Down	IAA21
Sme2.5_09216.1_g00004.1	113.5	324.0	319.8	-1.5	-1.5	Down	cytochrome P450 83B1-like
Sme2.5_08551.1_g00002.1	3.0	27.5	14.5	-3.2	-2.3	Down	probable indole-3-acetic acid-amido synthetase GH3.1
Sme2.5_03707.1_g00004.1	107.8	232.8	264.8	-1.1	-1.3	Down	small auxin-up protein 58
Genes related to gibberellin and cytokinin							
Sme2.5_25147.1_g00001.1	196.0	468.8	436.8	-1.3	-1.2	Down	serine/threonine-protein kinase SAPK2
Sme2.5_06317.1_g00002.1	16.5	41.0	38.3	-1.3	-1.2	Down	gibberellin 3-beta-dioxygenase
Sme2.5_02777.1_g00005.1	23.0	2.5	4.0	3.2	2.5	Up	cytokinin hydroxylase
Sme2.5_03751.1_g00003.1	25.8	0	0	---	---	Up	cytokinin hydroxylase
Sme2.5_04568.1_g00002.1	14.0	436.3	428.5	-5.0	-4.9	Down	cytokinin hydroxylase
Sme2.5_17618.1_g00001.1	1.0	9.3	6.3	-3.3	-2.6	Down	cytokinin hydroxylase
Sme2.5_03119.1_g00004.1	9.5	24.0	52.0	-1.3	-2.5	Down	cytokinin hydroxylase
Genes related to flower development							
Sme2.5_02912.1_g00001.1	10.5	0.8	4.5	3.9	1.2	Up	NAC domain-containing protein 100-like
Sme2.5_09643.1_g00001.1	133.8	47.0	37.3	1.4	1.8	Up	NAC domain-containing protein 21/22-like
Sme2.5_00374.1_g00015.1	301.5	88.5	49.5	1.8	2.6	Up	agamous-like MADS-box protein AGL80
Sme2.5_04102.1_g00005.1	69.3	438.0	166.8	-2.6	-1.3	Down	zinc finger protein CONSTANS-LIKE 2-like

Notes:

^*a*^ Average expression level means the normalized read counts of each transcript.

^*b*^ --- corresponds to genes specially expressed in PP05, PnP05 or GnP05.

### Validation of differential gene expression data by qRT-PCR

To validate the accuracy and reproducibility of the transcriptome analysis, nine genes (as shown in S1 Table) were selected randomly for qRT-PCR comparison of their expression levels between parthenocarpic and non-parthenocarpic eggplant samples. For the selected gene population, positive correlations between the fold changes determined by RNA-seq and qRT-PCR, the R^2^ values of PP05 *vs* PnP05 and PP05 *vs* GnP05 were 0.96 and 0.95, separately (S1 Table), thereby indicating that our RNA-seq data were credible.

## Discussion

Transcriptome analysis is of growing importance, as it helps comprehensive investigation of altered gene expression of genetic variants, and provides insights into the molecular basis for specific biological processes. To better understand the molecular mechanism of eggplant parthenocarpy, which is a desirable agronomic trait in eggplant breeding, transcriptomic profiles of eggplant parthenocarpic and non-parthenocarpic lines were screened. A large number of eggplant unigenes (38,925), as well as 506 common DEGs were identified and analyzed. As we know, this is the first comparative transcriptomic analysis in eggplant regarding to natural parthenocarpy.

The plant hormone auxin plays a critical role in regulating various aspects of plant growth and development, including the fruit set process. Contributions of auxin in regulation of parthenocarpy have therefore been extensively studied for many years. Exogenous application of auxin and modulation of key genes in auxin pathways, have been demonstrated to trigger parthenocarpy [13, 37]. In the present study, several DEGs related to auxin biogenesis and signaling were identified. Two calcium-binding protein PBP1 were up-regulated in PP05. In *Arabidopsis*, calcium-binding protein PBP1 is up-regulated by auxin, and stimulates the activity of PINOID (PID) protein kinase, which phosphorylates PIN (PIN-FORMED) proteins [38]. Besides, a serine/threonine-protein phosphatase 6 regulatory ankyrin repeat subunit B (PP6-ARS-B) was found down-regulated in PP05. This regulatory subunit may serve as a component of functional PP6 holoenzyme [39], which counteracts to the action of PID kinase to dephosphorylate PIN proteins in *A*. *thaliana* [40]. In view of the evidences that the antagonistic effects of protein phosphatase and PID proteins on PIN phosphorylation, which mediates PIN polarity and directs auxin distribution [41, 42], the enhanced expression of calcium-binding protein PBP1 and the decreased transcription of PP6-ARS-B in PP05 flower buds may alter the auxin flows and promote fruit development.

The transcription factor E2FB plays a role in regulating auxin-dependent cell division. The accumulation and stability of E2FB are positively regulated by auxin [43]. *Arabidopsis* cells overexpression of E2FB shows acceleration of cell division and reduction of cell cycle [43]. The ectopic expression of *Arabidopsis* E2FB in tomato leads to significantly earlier germination and flowering than the wild-type, generates more and bigger fruits, as well as significantly higher fruit yield [44]. Here, an E2FB homologue (Sme2.5_14564.1_g00002.1) was up-regulated in PP05. It was speculated that the E2FB might be involved in the auxin-modulated flower growth and fruit development in eggplant. Besides, F-box proteins constitute a large family in eukaryotes and are involved in various cellular processes, and one of these F-box proteins, TIR1 (Transport inhibitor response 1) is related to auxin perception [45]. In this study, a total of 226 genes were predicted to be F-box protein coding sequences, and 7 of them were found to be differentially regulated in PP05. Besides, two of these differential F-box proteins contain Leucine-rich repeats (LRR) domain, which is responsible for binding of auxin [46, 47], suggesting that these two F-box proteins may be involved in auxin signaling. Considering that members of the Aux/IAA family proteins contribute negatively to auxin signal transduction [48], and inhibition of AUX/IAA protein IAA9 in tomato triggers fruit development before fertilization and leads to parthenocarpy [49], down-regulation of an Aux/IAA homologue (IAA21) might facilitate auxin signaling in PP05 and parthenocarpy in eggplant.

In addition, we detected a gene encoding a protein similar to cytochrome P450 83B1 (CYP83B1) significantly down-regulated in PP05. CYP83B1 is an important enzyme at the metabolic branch point in indole-3-acetic (IAA, the major type of auxin) and indole glucosinolate biogenesis, mutation in CYP83B1 leads to the increase of IAA, whereas overexpression of CYP83B1 leads to a reduced IAA phenotype in *Arabidopsis* [50]. Other two DEGs encoding auxin-responsive proteins (indole-3-acetic acid-amido synthetase GH3.1 and small auxin-up protein 58) were identified down-regulated in PP05. Notably, the decrease of the GH3.1 might cause the release of auxin. Since members of the GH3 gene family encoding IAA-amino synthetases regulate (prevent) free IAA accumulation and down-regulating auxin signaling [51, 52]. Thus, the down-regulation of the CYP83B1 and GH3.1 homologs may result in an IAA increased phenotype in PP05 flower buds.

Besides, signaling of gibberellin (GA) and cytokinin (CTK), two major regulators of plant growth, is essential for fruit growth [53]. Accumulated data suggest that GA is responsible for the cell expansion and CTK for the cell division during fruit development [54]. Exogenous applications of GA and CTK to the flowers before fertilization are able to stimulate parthenocarpic growth in some species (*i*.*e*. pea) [55]. Besides, the interactions between these hormones are also important for fruit development. In tomato, parthenocarpic fruit-set induced by auxins has been demonstrated to be mediated, in part, by GA [56]. And a recent study based on the interpretation of transcriptome profiles of pollination-dependent and parthenocarpic ovaries in tomato, emphasize again, the integral roles of auxin and GA in fruit set [16]. In addition, enhanced GA and auxin biogenesis is required partly for the CTK-induced parthenocarpic tomato development [57]. In the present study, we found that the expression of a serine/threonine-protein kinase SAPK2, which is highly similar to PKABA1, a negative regulator of GA action [58], was down-regulated; whereas a GA biosynthesis enzyme gibberellin 3-beta-dioxygenase [59] was also repressed in PP05. Transcripts associated with CTK were also differentially expressed in PP05 flower buds, with an increase of two cytokinin hydroxylases and decrease of three cytokinin hydroxylases, which is involved in the biogenesis of an isoprenoid CTK *trans*-Zeatin [60]. It is hypothesized that the homeostasis of GA and CTK might be altered in PP05, and these changes might be necessary to promote cell expension/division in flower buds and trigger parthenocarpy.

In plants capable of displaying parthenocarpy, the fruit set and development occur without fertilization, and probably the genetic program for parthenocarpy has been set up already during early stages of flower development before anthesis [28]. Therefore, there should be some differences between parthenocarpic and non-parthenocarpic flowers development. The parthenocarpic plant constructed based on ovary-specific expression of *iaaM* gene exhibits similar morphology to the wild-type, but with higher levels of auxin in female gametophyte and an early enlargement of ovary [61]. In the present study, a subset of transcription factors (TFs) associated with flower development were identified differentially expressed. Among these identified TFs, two NAC domain-containing proteins were significantly up-regulated in PP05. Basically, NAC domain-containing TFs are considered as important plant regulators in coordinating developmental programs and stress perception [62]. In *Arabidopsis*, NAC family protein VOZ1 (vascular plant one zinc finger 1) and VOZ2 were identified to positively regulate the flowering time, the VOZ1 VOZ2-deficient mutant showed a late flowering phenotype [63]. The up-regulation of an AGAMOUS-like MADS-box protein was observed in PP05. It belongs to the MADS-box TF family, which has been shown to play a significant role in the regulation of flower and fruit development [64]. Roy Choudhury et al. [65] reported the involvement of a banana AGAMOUS-like MADS-box gene *MA-MADS5* in the floral reproductive organ development and fruit ripening, repression of the *MA-MADS5* homolog in tobacco by expressing the antisense *MA-MADS5* resulted in delayed flowering and reduced fruit size. Thus, the up-regulation of these TFs may promote the growth of flower buds and lead to precocious potential of PP05. The nuclear protein *CONSTANS* (CO) is a TF that plays a central role in promoting the photoperiodic flowering in *Arabidopsis* by mediating the expression of downstream genes *FT* (FLOWERING LOCUS T) and *SOC1* (SUPPRESSOR OF OVEREXPRESSION OF CO 1) [66]. Whereas many members in the CONSTANS-LIKE (COL) gene family, like COL9 in *Arabidopsis* and OsCO3 in rice, regulate flowering time negatively by repressing the expression of *CO* and *FT*-like genes, respectively [67, 68]. According to our result, a COL homolog (Sme2.5_04102.1_g00005.1) showed lower expression level in PP05, relative to that in PnP05 and GnP05. This is consistent with our conclusion that PP05 may have setup a genetic program before anthesis for its precocious ability, which meets the requirement for early spring planting of eggplant natural parthenocarpic lines. These observations lead to an interesting assumption that the possible involvement of flowering regulation in eggplant natural parthenocarpy.

## Conclusions

In conclusion, the comprehensive transcriptome profiles of eggplant during flower growth were obtained in the present study. RNA-seq data from a collection of eggplant flower buds with or without natural parthenocarpic capacity provided us new tools for studying mechanisms of parthenocarpy. In total, the RNA deep sequencing generated > 46 million clean reads in each sample, and 506 DEGs potentially related to parthenocarpy were filtered from eggplants PP05 *vs* PnP05 + GnP05. Many differentially transcribed sequences were predicted to be involved in the phytohormone signaling and flower development. Therefore, we proposed that, at least, auxin signaling synergies with other hormones, and regulation of flowering, might be necessary for the development of the natural parthenocarpic eggplant. Further investigation on the molecular functions of these candidates may provide new clues to parthenocarpic fruit set in eggplant.

## Supporting information

S1 Fig Eggplant flowers (A) and ovaries (B). Flowers and ovaries of eggplants PP05, PnP05 and GnP05 at the first day of anthesis, a centimeter ruler on the right side of each figure indicates the size of flowers and ovaries.
(TIF)

S2 Fig Classification of raw reads. (TIF)

S3 Fig Percent of reads mapped to reference genome regions. (TIF)

S4 Fig The analysis of alternative splicing (AS) events. The abbreviations of *y*-axis indicate 12 different types of AS events as follows: skipped exon (SKIP), approximate SKIP (XSKIP), multi-exon SKIP (MSKIP), approximate MSKIP (XMSKIP), intron retention (IR), approximate IR (XIR), approximate MIR (XMIR), alternative exon ends (AE), approximate AE (XAE), alternative 3' last exon (TTS), alternative 5' first exon (TSS), multi-IR (MIR). The *x*-axis indicates the numbers of AS events (log value).
(TIF)

S1 Table Genes and primers for validation of RNA-Seq results by qRT-PCR. (DOC)

S2 Table Summary of mapping results. (DOCX)

S3 Table Data of Pearson correlation between four replicates of each eggplant line. (XLSX)

S4 Table List of detailed information of genes significantly regulated in the PP05 compared to PnP05 and GnP05. (XLSX)

S5 Table The top 8 more represented GO terms of common DEGs in each main GO category. (DOC)

S6 Table The detailed information of top 10 more represented KEGG pathways. (DOC)
