# Supplementary figures and images for: Comparative transcriptome analysis provides insights into molecular mechanisms for parthenocarpic fruit development in eggplant (Solanum melongena L.)
Source: PLoS One. 2017 Jun 12;12(6):e0179491. doi: 10.1371/journal.pone.0179491 (PMC5467848; doi:10.1371/journal.pone.0179491)

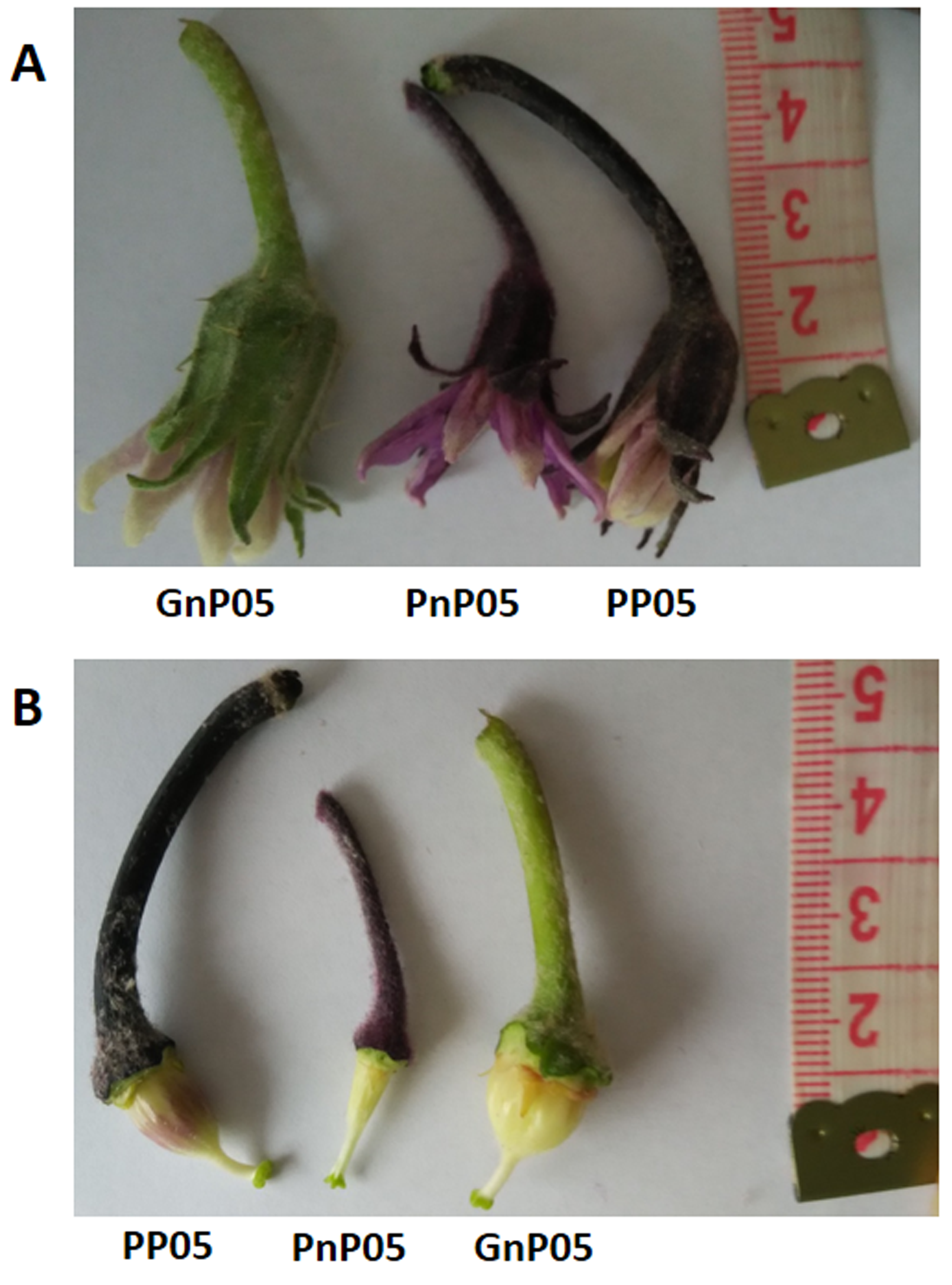

Supplement: S1 Fig — Flowers and ovaries of eggplants PP05, PnP05 and GnP05 at the first day of anthesis, a centimeter ruler on the right side of each figure indicates the size of flowers and ovaries. (TIF) [file pone.0179491.s001.tif]

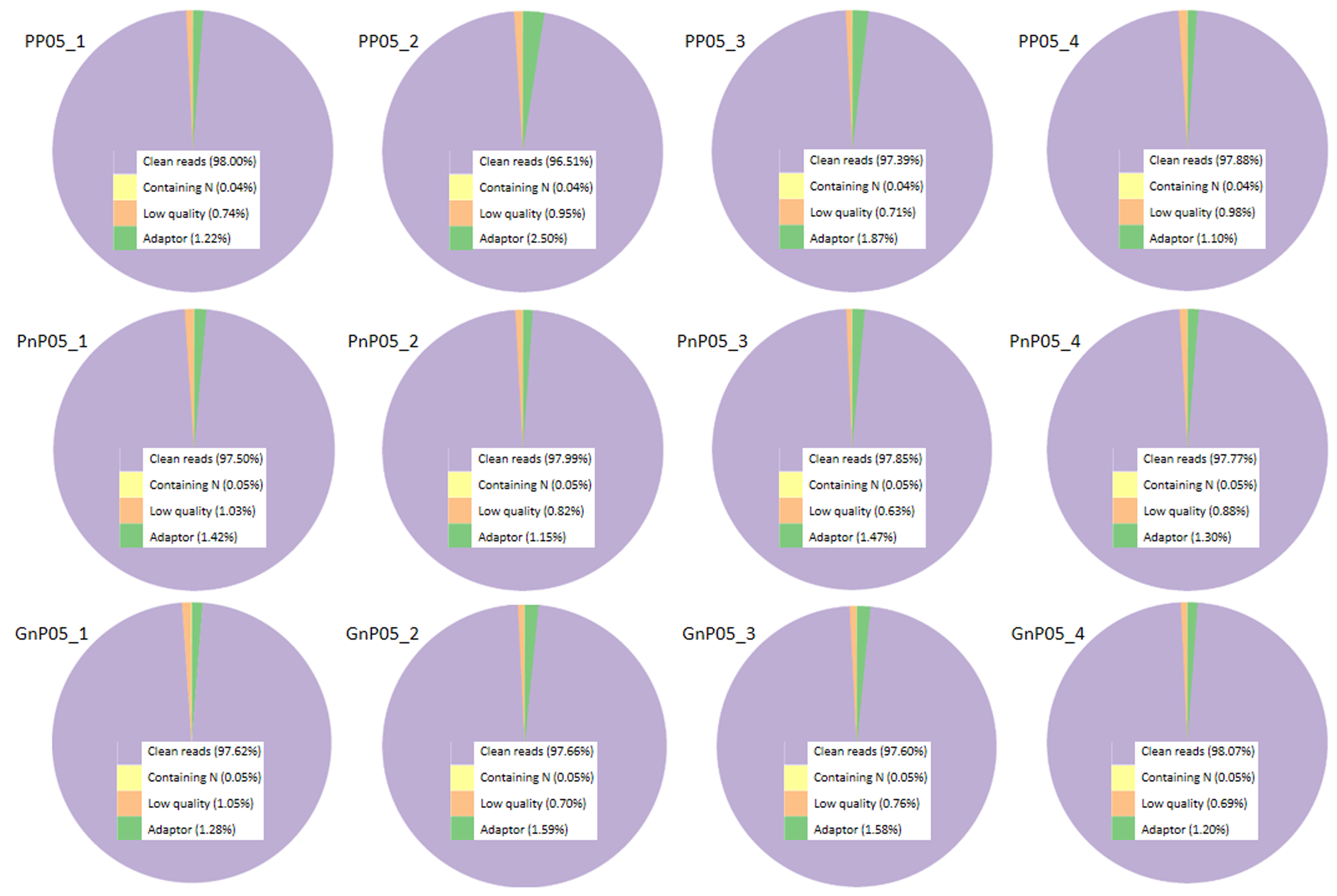

Supplement: S2 Fig — (TIF) [file pone.0179491.s002.tif]

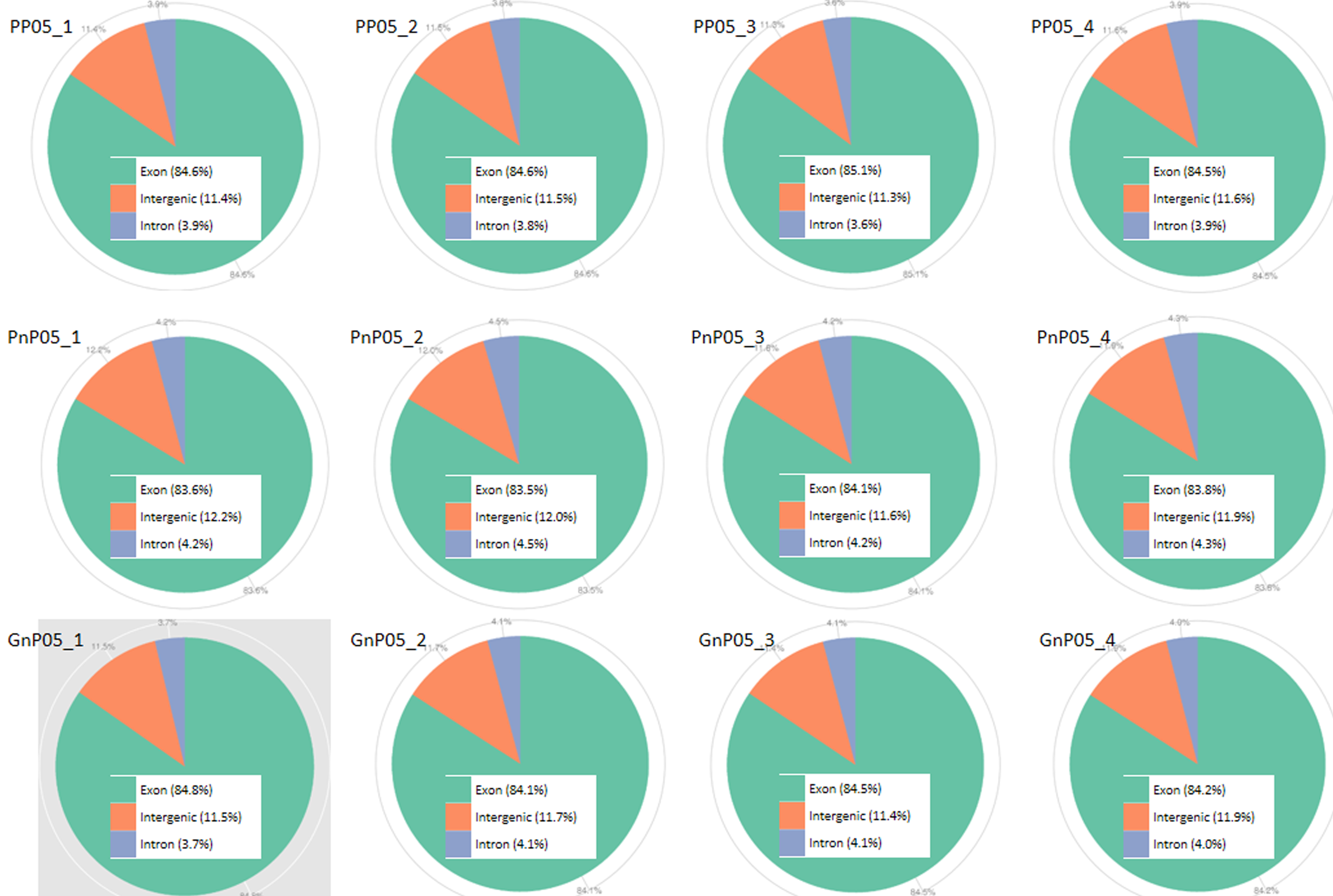

Supplement: S3 Fig — (TIF) [file pone.0179491.s003.tif]

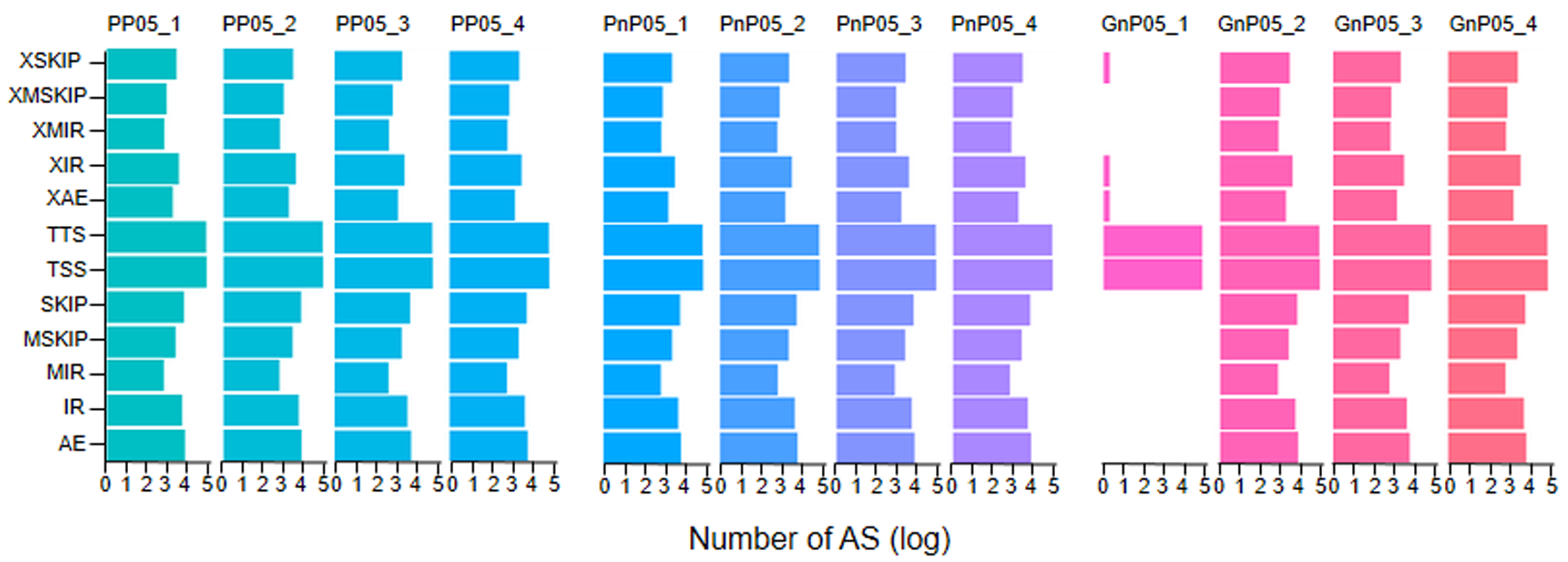

Supplement: S4 Fig — The abbreviations of y-axis indicate 12 different types of AS events as follows: skipped exon (SKIP), approximate SKIP (XSKIP), multi-exon SKIP (MSKIP), approximate MSKIP (XMSKIP), intron retention (IR), approximate IR (XIR), approximate MIR (XMIR), alternative exon ends (AE), approximate AE (XAE), alternative 3' last exon (TTS), alternative 5' first exon (TSS), multi-IR (MIR). The x-axis indicates the numbers of AS events (log value). (TIF) [file pone.0179491.s004.tif]
