# Supplementary material for: Comparative transcriptome analysis provides insights into molecular mechanisms for parthenocarpic fruit development in eggplant (Solanum melongena L.)
Source: PLoS One. 2017 Jun 12;12(6):e0179491. doi: 10.1371/journal.pone.0179491 (PMC5467848; doi:10.1371/journal.pone.0179491)
Supplement: S1 Table — (DOC) [file pone.0179491.s005.doc]

**Table S1.** Genes and primers for validation of RNA-Seq results by qRT-PCR

| Gene ID | Functional annotation | | Primers (5’ → 3’) | Length  (bp) | Fold change | | | |
| --- | --- | --- | --- | --- | --- | --- | --- | --- |
|  |  |  |  |  | PP05 *vs* PnP05 | | PP05 *vs* GnP05 | |
|  |  |  |  |  | RNA-Seq | qRT-PCR | RNA-Seq | qRT-PCR |
| Sme2.5_01401.1_g00004.1 | | DFR | F: CTAGCTGGAGCGACTTGGAC | 95 | 1.05 | 0.93 ± 0.04 | 3.82 | 7.72 ± 0.42 |
|  |  |  | R: TTCCCTTGCAGCCTTCTCTG |  |  |  |  |  |
| Sme2.5_01916.1_g00007.1 | | CYP97C11 | F: GGGCTGTGGTTCCATCTCTT | 70 | 0.99 | 0.98 ± 0.03 | 0.97 | 1.00 ± 0.06 |
|  |  |  | R: GCACATCTGCAAAAGACCCG |  |  |  |  |  |
| Sme2.5_03894.1_g00007.1 | | CINV1 | F: AGAGCTGGGAGAAAACGGTG | 60 | 0.01 | 0.01 ± 0.004 | 0.01 | 0.01 ± 0.001 |
|  |  |  | R: AACTTGCGGGCATCAATCCT |  |  |  |  |  |
| Sme2.5_06855.1_g00002.1 | | tryptophan synthase | F: TACGTCTTCACCCACCAAGC | 68 | 1.21 | 1.05 ± 0.06 | 0.88 | 1.08 ± 0.09 |
|  |  |  | R: CGGCGGCATCCATTGATTTT |  |  |  |  |  |
| Sme2.5_08608.1_g00002.1 | | elongation factor 1-alpha | F: CTGGTACAAGGGACCAACCC | 72 | 1.25 | 1.01 ± 0.01 | 0.97 | 0.65 ± 0.01 |
|  |  |  | R: GGCTTGTCTGATGGCCTCTT |  |  |  |  |  |
| Sme2.5_11939.1_g00002.1 | | actin-7 | F: CGTTGCCCAGAAGTCCTCTT | 62 | 0.97 | 0.81 ± 0.01 | 1.03 | 0.79 ± 0.01 |
|  |  |  | R: TCATGGATACCCGCAGCTTC |  |  |  |  |  |
| Sme2.5_16959.1_g00001.1 | | cis-zeatin O-glucosyltransferase | F: CATGCGCCTAGCGTCTTTTC | 60 | 1.64 | 1.18 ± 0.04 | 0.66 | 0.54 ± 0.04 |
|  |  |  | R: TGGATTTCACCCACGGAGTT |  |  |  |  |  |
| Sme2.5_25147.1_g00001.1 | | SAPK2 | F: AGGGACACCAGCTTACGTTG | 70 | 0.41 | 0.43 ± 0.05 | 0.45 | 0.50 ± 0.02 |
|  |  |  | R: AACATCTGCAACCTTCCCGT |  |  |  |  |  |
| Sme2.5_30391.1_g00001.1 | | xanthoxin dehydrogenase | F: CACTGGAGGTGCAAGTGGTA | 61 | 0.04 | 0.04 ± 0.003 | 0.02 | 0.09 ± 0.002 |
|  |  |  | R: CGCGCCATGATTGTGAAACA |  |  |  |  |  |
| Sme2.5_03686.1_g00005.1 | | tubulin gamma | F: GCTCTTTCTCTCCCTCTCGC | 71 | Internal control | | | |
|  |  |  | R: GCGATGAGGCAGTATCGGAA |  |  |  |  |  |
