# Supplementary material for: Comparative transcriptome analysis provides insights into molecular mechanisms for parthenocarpic fruit development in eggplant (Solanum melongena L.)
Source: PLoS One. 2017 Jun 12;12(6):e0179491. doi: 10.1371/journal.pone.0179491 (PMC5467848; doi:10.1371/journal.pone.0179491)
Supplement: S2 Table — (DOCX) [file pone.0179491.s006.docx]

| No. | Sample name | Total clean reads | Total mapped *^a^* | Multiple mapped *^b^* | Uniquely mapped *^c^* | Reads map to '+' *^d^* | Reads map to '-' *^d^* | Non-splice reads *^e^* | Splice reads *^e^* |
| --- | --- | --- | --- | --- | --- | --- | --- | --- | --- |
| 1 | PP05_1 | 58277782 | 48001790 | 1564137 | 46437653 | 23219773 | 23217880 | 28626798 | 17810855 |
| 2 | PP05_2 | 60525246 | 48846872 | 1669979 | 47176893 | 23588775 | 23588118 | 29251403 | 17925490 |
| 3 | PP05_3 | 55143180 | 45352336 | 1552628 | 43799708 | 21899845 | 21899863 | 26702299 | 17097409 |
| 4 | PP05_4 | 49119262 | 40014771 | 1277551 | 38737220 | 19366708 | 19370512 | 23800066 | 14937154 |
| 5 | PnP05_1 | 61456426 | 50652048 | 1686873 | 48965175 | 24485781 | 24479394 | 30873939 | 18091236 |
| 6 | PnP05_2 | 46924756 | 38985875 | 1221906 | 37763969 | 18891766 | 18872203 | 23489796 | 14274173 |
| 7 | PnP05_3 | 64580670 | 53269343 | 1709442 | 51559901 | 25794211 | 25765690 | 31813007 | 19746894 |
| 8 | PnP05_4 | 51941728 | 43271514 | 1397136 | 41874378 | 20948731 | 20925647 | 25957935 | 15916443 |
| 9 | GnP05_1 | 52944902 | 43618839 | 1475764 | 42143075 | 21076825 | 21066250 | 25830482 | 16312593 |
| 10 | GnP05_2 | 60462982 | 50806734 | 1643706 | 49163028 | 24573477 | 24589551 | 30030327 | 19132701 |
| 11 | GnP05_3 | 59656812 | 49220580 | 1540849 | 47679731 | 23836597 | 23843134 | 29316168 | 18363563 |
| 12 | GnP05_4 | 55900718 | 47052540 | 1580797 | 45471743 | 22729161 | 22742582 | 27643598 | 17828145 |

Table S2. Summary of mapping results.

Notes:

*^a^* Total mapped: number of clean reads mapped onto the reference genome.

*^b^* Multi-mapped: number of clean reads mapped to two or more sites on the reference genome.

*^c^* Uniquely mapped: number of clean reads mapped to only one site on the reference genome.

*^d^* Reads map to ‘+’ and ’-’: numbers of clean reads mapped to the positive strand and negative strand of the reference genome, respectively.

*^e^* Non-splice reads: numbers of clean reads mapped to only one exon on the reference genome; splice reads: number of junction reads span two exons on the reference genome.
