# Supplementary material for: Comparative transcriptome analysis provides insights into molecular mechanisms for parthenocarpic fruit development in eggplant (Solanum melongena L.)
Source: PLoS One. 2017 Jun 12;12(6):e0179491. doi: 10.1371/journal.pone.0179491 (PMC5467848; doi:10.1371/journal.pone.0179491)
Supplement: S5 Table — (DOC) [file pone.0179491.s009.doc]

**Table S5.** The top 8 more represented GO terms of common DEGs of each main GO category.

| GO term | GO term annotation | DEG number | Adjusted *P*-value |
| --- | --- | --- | --- |
| **Biological process (Up-regulated)** | | | |
| GO:0015074 | DNA integration | 8 | 0.000106 |
| GO:0006259 | DNA metabolic process | 39 | 0.000766 |
| GO:0006278 | RNA-dependent DNA replication | 22 | 0.002288 |
| GO:0006260 | DNA replication | 25 | 0.003749 |
| GO:0090304 | nucleic acid metabolic process | 58 | 0.010537 |
| GO:0006139 | nucleobase-containing compound metabolic process | 62 | 0.01984 |
| GO:0046483 | heterocycle metabolic process | 65 | 0.040093 |
| GO:0006807 | nitrogen compound metabolic process | 68 | 0.045079 |
| **Biological process (Down-regulated)** | | | |
| GO:0090304 | nucleic acid metabolic process | 40 | 1.3E-09 |
| GO:0006259 | DNA metabolic process | 28 | 2.01E-09 |
| GO:0006139 | nucleobase-containing compound metabolic process | 44 | 2.49E-09 |
| GO:0006260 | DNA replication | 14 | 5.56E-09 |
| GO:0034641 | cellular nitrogen compound metabolic process | 47 | 7.16E-09 |
| GO:0046483 | heterocycle metabolic process | 47 | 7.75E-09 |
| GO:0006725 | cellular aromatic compound metabolic process | 48 | 1.55E-08 |
| GO:0034645 | cellular macromolecule biosynthetic process | 30 | 1.7E-08 |
| **Cellular component (Up-regulated)** | | | |
| GO:0043227 | membrane-bounded organelle | 17 | 0.03073 |
| GO:0043231 | intracellular membrane-bounded organelle | 17 | 0.031223 |
| GO:0016020 | membrane | 46 | 0.03337 |
| GO:0043232 | intracellular non-membrane-bounded organelle | 14 | 0.039231 |
| GO:0043228 | non-membrane-bounded organelle | 14 | 0.039231 |
| GO:0044425 | membrane part | 21 | 0.044122 |
| GO:0005840 | ribosome | 8 | 0.049577 |
| GO:0005575 | cellular_component | 78 | 0.049917 |
| **Cellular component (Down-regulated)** | | | |
| GO:0005886 | plasma membrane | 7 | 0.002112 |
| GO:0005622 | intracellular | 30 | 0.005987 |
| GO:0044424 | intracellular part | 28 | 0.006911 |
| GO:0043226 | organelle | 21 | 0.008676 |
| GO:0043229 | intracellular organelle | 21 | 0.009293 |
| GO:0005634 | nucleus | 9 | 0.017945 |
| GO:0005623 | cell | 37 | 0.027227 |
| GO:0044464 | cell part | 37 | 0.027227 |
| **Molecular function (Up-regulated)** | | | |
| GO:0003964 | RNA-directed DNA polymerase activity | 22 | 0.001061 |
| GO:0016779 | nucleotidyltransferase activity | 25 | 0.001236 |
| GO:0034061 | DNA polymerase activity | 22 | 0.001341 |
| GO:0003723 | RNA binding | 25 | 0.002058 |
| GO:0016772 | transferase activity, transferring phosphorus-containing groups | 34 | 0.006704 |
| GO:0043565 | sequence-specific DNA binding | 9 | 0.038973 |
| GO:0030234 | enzyme regulator activity | 10 | 0.044142 |
| GO:0003676 | nucleic acid binding | 58 | 0.049118 |
| **Molecular function (Down-regulated)** | | | |
| GO:0003676 | nucleic acid binding | 28 | 1.07E-13 |
| GO:0016779 | nucleotidyltransferase activity | 15 | 5.99E-09 |
| GO:0003723 | RNA binding | 16 | 6.79E-09 |
| GO:0034061 | DNA polymerase activity | 14 | 1.89E-08 |
| GO:0003964 | RNA-directed DNA polymerase activity | 14 | 2.92E-08 |
| GO:0016772 | transferase activity, transferring phosphorus-containing groups | 29 | 3.62E-05 |
| GO:0097159 | organic cyclic compound binding | 63 | 0.000371 |
| GO:0008270 | zinc ion binding | 10 | 0.000427 |
