# Supplementary material for: Comparative transcriptome analysis provides insights into molecular mechanisms for parthenocarpic fruit development in eggplant (Solanum melongena L.)
Source: PLoS One. 2017 Jun 12;12(6):e0179491. doi: 10.1371/journal.pone.0179491 (PMC5467848; doi:10.1371/journal.pone.0179491)
Supplement: S6 Table — (DOC) [file pone.0179491.s010.doc]

**Table S6.** The detailed information of top 10 more represented KEGG pathways.

| KEGG pathway | | KEGG pathway annotation | DEG number | | | Adjusted *P*-value |
| --- | --- | --- | --- | --- | --- | --- |
| **Up-regulated** | | | | | | |
| sly01110 | Biosynthesis of secondary metabolites | | | 9 | 0.000005 | |
| sly00909 | Sesquiterpenoid and triterpenoid biosynthesis | | | 3 | 0.000004 | |
| sly01100 | Metabolic pathways | | | 9 | 0.000252 | |
| sly00350 | Tyrosine metabolism | | | 2 | 0.004100 | |
| sly00630 | Glyoxylate and dicarboxylate metabolism | | | 2 | 0.008256 | |
| sly00360 | Phenylalanine metabolism | | | 2 | 0.033366 | |
| sly00603 | Glycosphingolipid biosynthesis - globo series | | | 1 | 0.030827 | |
| sly00940 | Phenylpropanoid biosynthesis | | | 2 | 0.037980 | |
| sly00591 | Linoleic acid metabolism | | | 1 | 0.047603 | |
| sly00950 | Isoquinoline alkaloid biosynthesis | | | 1 | 0.047525 | |
| **Down-regulated** | | | | | | |
| sly01110 | Biosynthesis of secondary metabolites | | | 9 | 0.000005 | |
| sly00940 | Phenylpropanoid biosynthesis | | | 5 | 0.000005 | |
| sly01100 | Metabolic pathways | | | 11 | 0.000006 | |
| sly00592 | alpha-Linolenic acid metabolism | | | 3 | 0.000072 | |
| sly00945 | Stilbenoid, diarylheptanoid and gingerol biosynthesis | | | 2 | 0.002096 | |
| sly00941 | Flavonoid biosynthesis | | | 2 | 0.002540 | |
| sly00460 | Cyanoamino acid metabolism | | | 2 | 0.002828 | |
| sly01200 | Carbon metabolism | | | 3 | 0.004448 | |
| sly00630 | Glyoxylate and dicarboxylate metabolism | | | 2 | 0.004745 | |
| sly00710 | Carbon fixation in photosynthetic organisms | | | 2 | 0.005491 | |
